# Long-term combined organic manure and chemical fertilizer application enhances aggregate-associated C and N storage in an agricultural Udalfs soil

**DOI:** 10.1371/journal.pone.0276197

**Published:** 2023-02-13

**Authors:** Shaojun Qiu, Cheng Hu, Donghai Liu, Shuanglai Liu, Shicheng Zhao, Xinpeng Xu, Ying Zhao, Ping He, Wei Zhou

**Affiliations:** 1 Key Laboratory of Plant Nutrition and Fertilizers, Ministry of Agriculture and Rural Affairs; Institute of Agricultural Resources and Regional Planning, Chinese Academy of Agricultural Sciences, Beijing, China; 2 Institute of Plant Protection, Soils and Fertilizers, Hubei Academy of Agricultural Sciences, Wuhan, China; 3 College of Resources and Environmental Engineering, Ludong University, Yantai, China; University of Minnesota, UNITED STATES

## Abstract

Little information is known on whether carbon (C) and nitrogen (N) immobilization is synchronized in different sizes of aggregates under different agricultural management practices. Carbon and N concentrations and the C/N ratios in different sizes of aggregates down to 40 cm depth were determined after long-term application of chemical fertilizers combined with manure or without manure in a wheat-rice cropping system. Manure application usually produced significantly (*P < 0*.*05*) higher C and N concentrations and lower C/N ratios in bulk soil and in different sizes of aggregates down to 20 cm depth than the other treatments, and the 1.5 MNPK treatment at 0–10 cm depth had the highest SOC concentration of 26.3 g/kg. The C and N concentrations in bulk soil and all aggregate fractions decreased markedly with increasing soil depth. Among water stable aggregates in all soil depths, the highest C (48.2–66.4%) and N (47.8–68.3%) concentrations as a percentage of SOC were found in the small macroaggregates (2000–250 μm, SM). Manure application significantly (*P < 0*.*05*) increased the mass and C and N concentrations of SM and SM fractions down to 20 depth. The mean C/N ratios of silt-clay within large and small macroaggregates (inter-SC) were 1.57 and 1.46 units lower than those of silt-clay particles, respectively, indicating that inter-SC had relative high N availability. Moreover, the C and N content of SM down to 40 cm depth tended to saturation with increasing C input rate. Overall, manure application effectively improved soil structure, SM were the dominant particles involved in soil C and N storage, and inter-SC were the main particles responsible for N availability.

## Introduction

Soil organic matter (SOM) contains twice to triple organic C than global vegetation and the atmosphere, it plays an important role in the supply and immobilization of nutrients and the storage and loss of carbon, the distribution of soil organic carbon and nitrogen (SOC, SON) can effectively indicate the quantity and quality of SOM [1, 2]. Given the worldwide requirement for carbon neutrality, the immobilization of C and N in soils has been increasingly accepted to be an effective measurement in the quest to decrease global warming potential caused by C and reactive N emissions in soil [1, 3, 4]. Currently, physical protection of soil aggregate is an important mechanism for the long-term SOC stabilization [5, 6]. Moreover, increase in SOC content is always accompanied by soil N immobilization because the labile C drives N immobilization and transformation via microbial activities [7]. In agricultural soils, repeated application of chemical fertilizers combined with manures is a sustainable management practice to improve soil fertility and maintain high grain yields. However, the pathways of soil N transformation are much more complex than those of soil C. Therefore, understanding whether C and N immobilization in soil aggregates are synchronized is beneficial to explore the mechanisms of soil C and N storage over the long term.

Bulk soil is usually divided into a labile pool and a stable pool [8, 9]. The labile pool is composed of young, undecomposable or semi-decomposable SOM that responds sensitively to management practices and represents C and N cycling in the short term. The stable pool is mainly composed of old and decomposed SOM and represents the inherently stable C over the long term [8, 10].

The most commonly labile pool is particulate organic matter (POM). The original POM is derived mainly from plant residues or exogenous organic materials and it is weakly bound with soil minerals. The original POM is incorporated into soil aggregates as it decomposes, and POM is the nucleus of aggregates according to the aggregate hierarchical theory [11–14]. Macroaggregates (> 250 μm) are formed around the POM and then the decomposed POM is associated with silt-clay sized aggregates (< 53μm) via microorganisms, the macroaggregates can be divided into large macroaggregates (LM, > 2000 μm) and SM (250–2000 μm), and SM is further bonded together to form LM. Polymers of microbial origin provide cohesion between the structural components of aggregates and form microaggregates (250–53μm) within macroaggregates. Microbial activity decreases gradually as the C and energy resource is depleted during POM biodegradation and the macroaggregates become unstable because of the lack of polymers of microbial origin [15–17]. Macroaggregates have more absolute C content than microaggregates but microaggregates sequester C to become more stable in the long term [8]. However, chemical fertilizer N in agricultural soils is involved in soil internal N cycling after it has dissolved in the soil solution or adsorbed onto soil clay minerals. The adsorption effect usually involves the silt-clay sized aggregates because silt-clay particles have the highest surface area and mineral ions of all different sizes of aggregates [18]. The application of manure and crop residues not only promotes the formation of aggregates but also increases N immobilization in soil particles due to their inherent high content of POM. Based on the characteristics of C and N immobilization in aggregates after the application of different fertilizers, there is heterotrophic placement of the C and N interactions between C decomposition from large fraction size (POM) and free-N in soil solution or immobilized-N in small fraction size (silt-clay). Thus, it is valuable to clarify the dominant or transitional particles for N immobilization associated with C storage within the aggregates.

The application of different chemical or organic fertilizers can affect soil C and N cycling or status in different soil pools and their C/N ratios. Low C/N ratios (< 20:1) of exogenous substrates will promote net N mineralization [19], while higher C/N ratios (> 30:1) will lead to transient competition for N between soil microorganisms and crops and increase soil N immobilization. The C/N ratio of POM in different size of aggregates reflects the quality of plant residue and their degree of decomposability, and the C/N ratio in the stable pools is a stabilizer for the C/N ratio of the bulk soil. Furthermore, differences in C/N ratios in soil pools can reflect N availability [20]. It is therefore important to explore the changes in C/N ratio in different sizes of aggregates when soil N immobilization is induced by application of chemical fertilizers or manures.

The Jianghai plain in Hubei province is the important grain base in central China, where the main two crops in a year are spring wheat and middle rice. Soil water-drought rotation further increases the complexity of SOM storage. When soil is flooded, soil structure is prone to collapse and is exposed to more nutrients, increasing soil C and N availability and the opportunity for soil particles to bind with exogenous C and N; When the soil is dried, dispersed soil particles are more easily exposed to soil oxygen and increase SOC mineralization. In addition, removal of aboveground biomass and increased soil disturbance by frequent tillage in agricultural soils further increases soil C and N variability. To avoid short-term changes in soil C and N storage due to soil heterogeneity and soil management practices, the long-term soil fertility experiments under different fertilization practices can provide the valuable information about SOM storage. The aims of the current study were therefore to (1) explore the C and N changes in different size classes of aggregates after long-term different fertilization practices and (2) elucidate the change in C/N ratio in different size classes of aggregates after long-term different fertilization practices. We hypothesized that (1) SM was the main particles for soil C and N storage according to water stable aggregates (WSA) hierarchy theory, and (2) free-SC had the relative high N availability given that the lowest occlusion among all the silt-clay particles.

## Materials and methods

### Site

The long-term wheat-rice rotation experiment is located in Wuhan city (30°37’N, 114°20’E), Hubei province, central China, and started in 1981. This region belongs to the transitional climate zone from northern subtropical to middle subtropical with an annual average temperature and precipitation of 16.4°C and 1300 mm. The soil is yellow-brown paddy soil with a clay loam texture classified as Udalfs (USDA soil classification). The physicochemical properties from 0–20 cm depth in 1981 was a pH of 6.3, a SOC concentration of 15.9 g kg^−1^, and a total N concentration of 1.8 g kg^−1^, total P concentration of 1.0 g kg^−1^, total K concentration of 30.2 g kg^−1^, alkali-hydrolyzable N concentration of 150.7 mg kg^−1^, Olsen-P concentration of 5.0 mg kg^−1^, and NH_4_Ac-K concentration of 98.5 mg kg^−1^.

### Experimental design

The experiment was a randomized complete block with three replicates and comprised four treatments: (1) control (CK) with no fertilizer or manure application, (2) optimum chemical fertilizer application (NPK), (3) optimum manure combined with chemical fertilizers (MNPK), (4) excessive fertilizer application with 1.5 times the manure rate of treatment (3) (1.5MNPK). Each plot was 5 m × 8 m with an area of 40 m^2^. Fertilizer sources were urea, NH_4_H_2_PO_4_, KCl and pig manure and the fertilizer rates are shown in Table 1. The N in NH_4_H_2_PO_4_ was included in the total N rate, the P and K fertilizers and manure were applied as basal fertilizers in each season crop. In the wheat season, N was applied in a ratio of 2:1:1 at basal, seedling and jointing stages. In the rice season, N was applied in a ratio of 4:2:2 at basal, tillering and booting stages. Wheat was sown at early November and harvested in late May of the following year. Rice was transplanted at early June and harvested in the middle of October.

**Table 1:** Fertilizer rates in control (CK), chemical fertilizer (NPK), and the combination of manure and chemical fertilizer (MNPK, 1.5MNPK) treatments for each crop.

Treatment	Wheat season	Rice season	Pig manure per season		
	N:P:K (kg ha^-1^)	N:P:K (kg ha^-1^)	Rate (t ha^-1^)	Manure C (kg ha^-1^)	Manure N (kg ha^-1^)
CK	0:0:0	0:0:0	0		
NPK	60:13:50	90:20:75	0		
MNPK	60:13:50	90:20:75	11.25	737.6	104.4
1.5MNPK	60:13:50	90:20:75	16.87	1106.4	156.6

After rice harvest in 2015 a composite sample of five soil cores down to 40 cm soil depth was taken from each treatment following a serpentine pattern and each soil sample was divided into 0–10, 11–20 and 21–40 cm soil depths for analysis of aggregate C and N. The bulk densities at each of these soil depths were 1.20, 1.32, and 1.45 g cm^-3^, respectively, in the control; 1.19, 1.27 and 1.47 g cm^-3^ in the NPK treatment; 1.02, 1.20 and 1.43 g cm^-3^ in the MNPK treatment; and 1.01, 1.13 and 1.42 g cm^-3^ in the 1.5MNPK treatment.

### Aggregate fractionation

Soil aggregate separation was conducted by wet sieving and density fractionation with heavy liquid according to literatures reports [8, 9, 21]. According to Brown et al. [9], WSA were classified with wet sieving into four size classes, namely LM (> 2000 μm), SM (2000–250 μm), free microaggregates (free-m) (250–53 μm), and free silt-clay (free-SC) (< 53 μm). Briefly, 80.0 g of air-dried soil was placed on a 2000-μm sieve and submerged in deionized water for 5 min at room temperature and separated with a 3-cm up and down movement 50 times over a period of 2 min. During the submersion process the floating material was removed with a net.

Microaggregates within macroaggregates (inter-m) in LM or SM were further isolated using the method of Six et al. [22]. Briefly, ≤ 15.0 g of oven-dried LM or SM were transferred onto a screen with a 250-μm mesh and transparent plastic wall. The screen was shaken in running water with 50 glass beads (4-mm diameter) until the water became clarified. The < 250 μm soil slurry continued to pass through the 53-μm mess screen by wet sieving was the water-stable aggregate fraction. The separated materials (> 250, 250–53 and < 53 μm) were coarse POM plus sand (cPOM + sand), inter-m, and silt-clay within macroaggregates (inter-SC).

Inter-m particles comprise three fractions: fine particulate organic matter (fPOM), fine intra particulate organic matter (fiPOM) and intra silt-clay particle (intra-SC). Firstly, 3–5 g of oven-dried inter-m were isolated to obtain the fPOM using sodium polytungstate at a density of 1.85 g cm^-3^ at a ratio of 1:3 (soil:liquid, w/v) and the residue was shaken for 12 h with 0.5% sodium hexametaphosphate at a ratio of 1:3 (soil:liquid, w/v). The dispersed slurry passed through a 53-μm sieve, and both particle sizes > 53 μm and < 53 μm were fiPOM + sand and intra-SC particles.

Finally, soil particles cPOM + sand and fiPOM + sand were isolated to remove sand using sodium polytungstate at a density of 2.3 g cm^-3^ at a ratio of 1:3 (soil:liquid, w/v). The floating particles were cPOM and fiPOM, respectively.

All soil particles recovered by sodium polytungstate fractionation were washed 7–10 times with deionized water on a 20-μm mesh using a vacuum filtration device. At each fractionation step above the separated soil particles were oven-dried at 60°C and then weighed on a balance with 0.01 or 0.0001 precision.

### Soil analysis

The C and N concentrations in the soil particles during aggregate fractionation and in the bulk soil passing through a 0.15-mm sieve were determined with a CN analyzer (Macrocube, Elementar, Hanau, Germany). Soil alkali-hydrolyzable N was determined using the Mason jar diffusion method [23]. Soil total P and K were digested in a nickel crucible with sodium hydroxide at 750°C. Soil Olsen-P was extracted with 0.5 M NaHCO_3_ and soil exchangeable K was extracted with 1 M NH_4_OAc. Soil total P and Olsen-P were determined using the molybdenum blue colorimetric method at a wavelength of 880 nm [24]. Soil total K and NH_4_Ac-K were determined by atomic absorption spectrophotometry [25]. Soil pH was determined in water (1:2.5). Soil bulk density was calculated using the dry weights and the volumes of sampled soil.

### Carbon input estimation

Carbon input was derived mainly from exogenous fertilizer C and returned crop residues C, the crop residues included belowground biomass and aboveground stubbles biomass. The calculated equation of crop residues C in the control and the chemical fertilizer treatments was shown as follows:

Wheat residues C input = [(GY / HI) × (30% / 70%) × 85% + (GY / HI) × (1-HI) × 18.3%] × (1–14%) × 0.399

Rice residues C input = [(GY / HI) × (30%+5.6%)] × (1–14%) × 0.418

Where GY, and HI are grain yield, and harvest index, respectively. The averaged GY in the control, NPK, MNPK, and 1.5MNPK treatments was 1.17, 2.39, 3.27, and 3.32 t/ha in winter wheat season as well as 4.20, 6.00, 6.43, and 6.47 t/ha in middle rice season, respectively. The HI values were 0.46 and 0.50 in winter wheat and middle rice seasons, respectively. 0.399 and 0.418 were average C concentration in wheat straw and rice straw. 14% was the average moisture of crop samples. In winter wheat season, 30% / 70% was the ratio of belowground biomass to aboveground biomass, 85% was the percentage of root biomass down to 40 cm depth in total root biomass, 18.3% was the percentage of aboveground stubble to aboveground biomass. In rice season, 30% was the percentage of belowground biomass to aboveground biomass, 5.6% was the percentage of aboveground stubbles to aboveground biomass [26, 27].

Carbon input in the MNPK and 1.5MNPK treatments was the sum of manure C and crop residues C, the manure C per season was shown in Table 1.

### Statistical analysis

Data are expressed on an oven-dried basis. One-way analysis of variance was conducted using the SPSS 24.0 software package and mean values of the variables among the treatments were compared using least significant difference at the 5% level. Partial correlations between aggregate C and N and their C/N ratios as respectively controlled C, N, and C/N ratio in bulk soil down to 20 cm soil depth was calculated by linear regression (PROC CORR) (see SI). The C and N contents or C/N ratios down to 40 cm soil depth in response to C input was determined using linear, quadratic, or logarithm functions using the SPSS 24.0 software package. The best function fitting the data according to the coefficient (R^2^) and the residual sum of squares was selected for calculation of the response curve.

## Results

**Fig 1:**
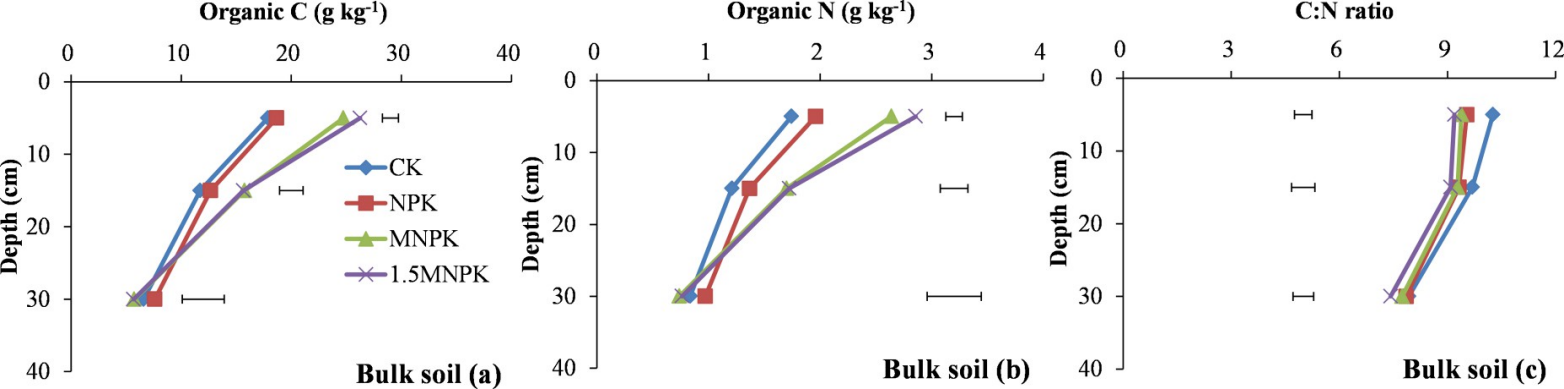
Soil organic C and N concentrations and their C/N ratios in bulk soil in the control (CK), chemical fertilizer (NPK), and combined manure and chemical fertilizer (MNPK, 1.5MNPK) treatments in the top 40 cm of the soil profile. Values at each soil depth are the mean of three replicates. The horizontal lines denote the least significant difference values at the 0.05 level (LSD0.05) among different treatments at each soil depth. LM, SM, free-m, and free-SC represent large macroaggregates (> 2000μm), small macroaggregates (2000–250 μm), microaggregates (53–250 μm), and silt-clay (< 53 μm); cPOM, inter-m, and inter-SC represent coarse particulate organic matter (POM), microaggregates within macroaggregates, and silt-clay within macroaggregates; fPOM, fiPOM, and intra-SC represent fine POM, fine intra POM and intra silt-clay within inter-m.

Manure application significantly (*P < 0*.*05*) increased SOC and SON concentrations compared with the other two treatments at 0–10 and 10–20 cm soil depths. Moreover, 1.5MNPK gave significantly (*P < 0*.*05*) higher SOC and SON concentrations than MNPK at 0–10 cm depth (Fig 1A and 1B). At 0–10 cm soil depth, the C and N concentrations in the 1.5MNPK treatment were 26.2 and 2.8 g/kg, and its C concentration was 1.1, 1.4, and 1.6 times higher than that in the MNPK, NPK, and control. Conversely, at 0–10 cm soil depth, the C/N ratio of bulk soil in the control was significantly (*P < 0*.*05*) higher than the fertilization treatments, and the highest C/N ratio was 10.3 in the control and the lowest was 9.2 in the 1.5MNPK treatment (Fig 1C).

**Fig 2:**
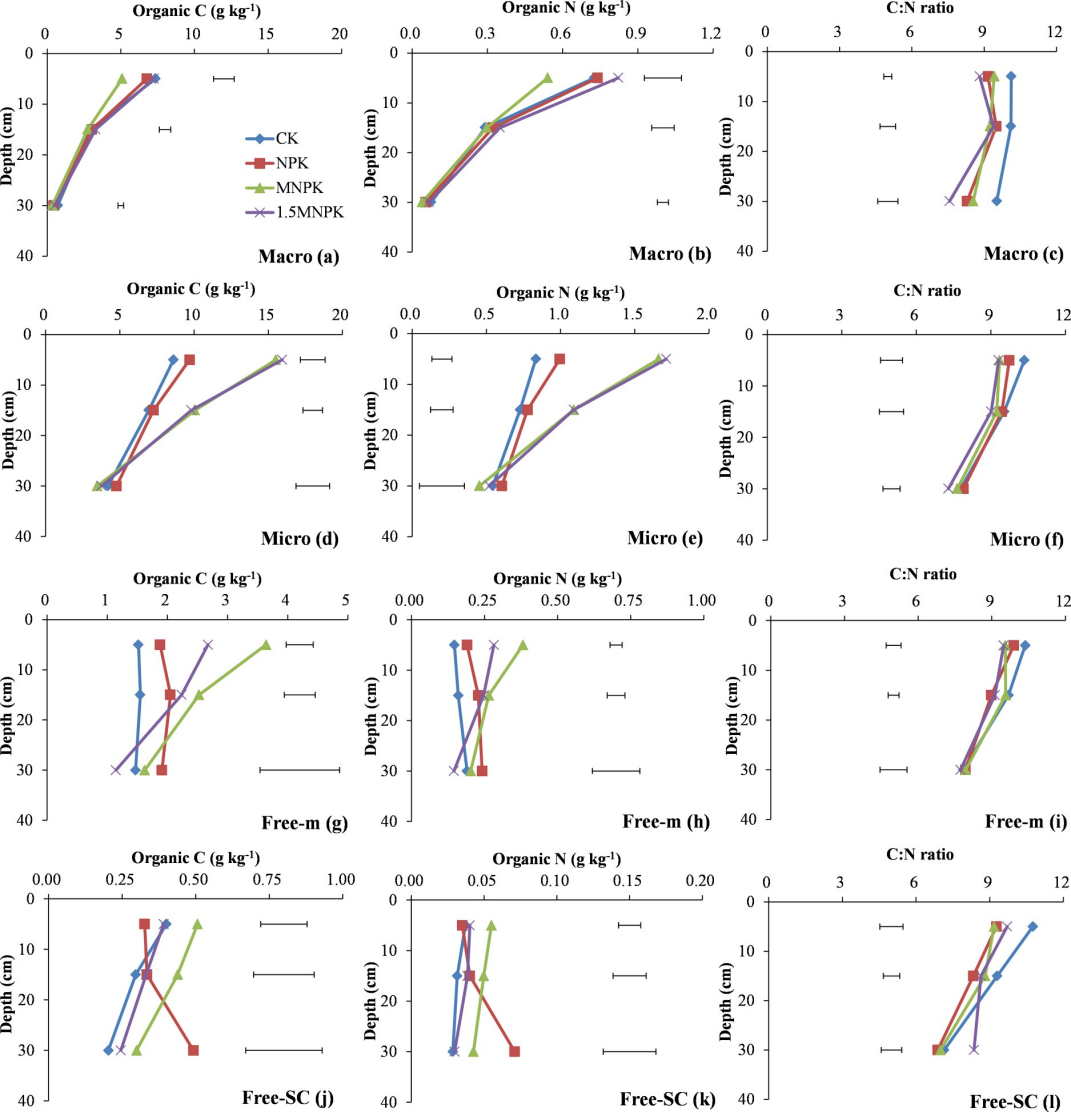
Change in organic C and N concentrations and their C/N ratios in water stable aggregate fractions (WSA) of control (CK), chemical fertilizer (NPK), and combined manure and chemical fertilizer (MNPK, 1.5MNPK) treatments in the top 40 cm of the soil profile. Values at each soil depth are the mean of three replicates. The horizontal lines denote the least significant difference values at the 0.05 level (LSD0.05) among different treatments at each soil depth. Symbols of aggregate fractions: see Fig 1 footnote.

MNPK significantly (*P < 0*.*05*) decreased C and N concentrations of LM than the other treatments in WSA fractions at 0–10 cm soil depth (Fig 2A and 2B), significantly (*P < 0*.*05*) increased those of SM and free-m than the NPK and control (Fig 2D, 2E, 2G and 2H), and significantly (*P < 0*.*05*) increased those of free-SC than the control (Fig 2J and 2K). 1.5MNPK had significantly (*P < 0*.*05*) lower C and N concentrations of free-m than the MNPK treatment and significantly (*P < 0*.*05*) higher than the NPK and control at 0–10 cm depth (Fig 2G and 2H). 1.5MNPK had significantly (*P < 0*.*05*) higher C and N concentrations of SM than the NPK and control at 0–10 and 10–20 cm depths (Fig 2D and 2E). At 20–40 cm depth, NPK had the highest C and N concentrations of free-SC among the four treatments, and was significantly (*P < 0*.*05*) higher than the control (Fig 2J and 2K). Besides, SM had the highest C and N concentrations among all the WSA fractions, with 48.2–66.4% and 47.8–68.3% of C and N concentrations in SM to bulk soil, respectively.

The control had the highest C/N ratio of all WSA fractions among the four treatments at 0–10 and 10–20 cm depths. Moreover, the control had significantly (*P < 0*.*05*) higher C/N ratio of the WSA fractions at 0–10 cm depth (Fig 2C, 2F, 2I and 2L) and that of LM at 10–20 and 20–40 cm depths compared with manure application (Fig 2C). 1.5MNPK gave significantly (*P < 0*.*05*) higher C/N ratio of free-SC than the other treatments at 20–40 cm depth (Fig 2L). The C/N ratio of all the WSA fractions ranged from 6.9 to 10.8.

**Fig 3:**
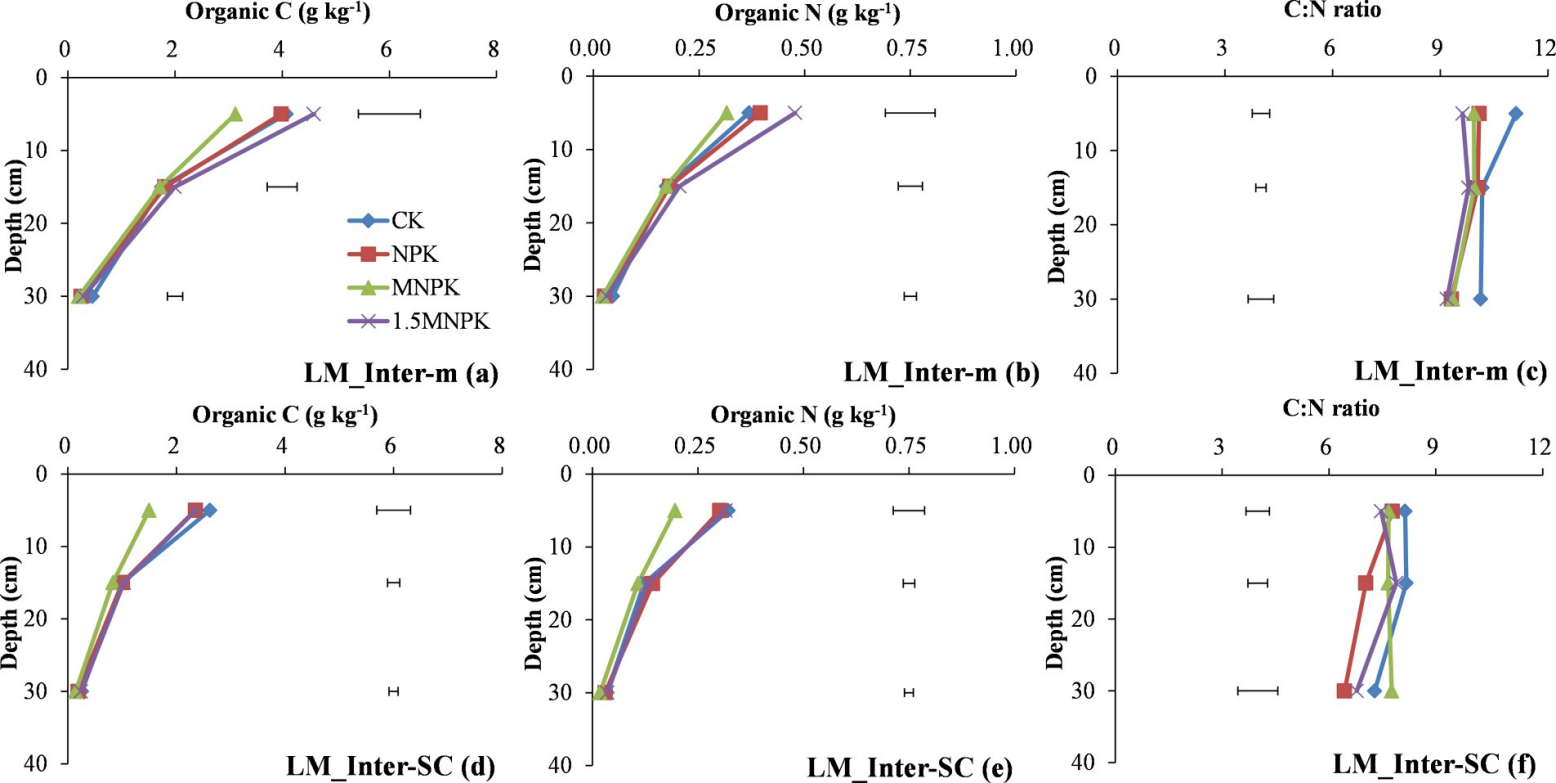
Change in organic C and N concentrations and their C/N ratios in large macroaggregate (LM) fractions of control (CK), chemical fertilizer (NPK), and the combined manure and chemical fertilizer (MNPK, 1.5MNPK) treatments in the top 40 cm of the soil profile. Values at each soil depth are the mean of three replicates. The horizontal lines denote the least significant difference values at the 0.05 level (LSD0.05) among different treatments at each soil depth. Symbols of aggregate fractions: see Fig 1 footnote.

The C and N concentrations of LM-inter-m and LM-inter-SC in the MNPK treatment were the lowest among the four treatments at each soil depth (Fig 3A, 3B, 3D and 3E). Moreover, there were significant (*P < 0*.*05*) differences between MNPK and 1.5MNPK treatments at 0–10 cm depth in the C and N concentrations of LM-inter-m and LM-inter-SC (Fig 3A, 3B, 3D and 3E).

At 0–10 cm depth, the C/N ratios of LM-inter-m and LM-inter-SC in the control were the highest among the four treatments (Fig 3C and 3F) and the C/N ratio of LM-inter-m in the control was significantly (*P < 0*.*05*) higher than in the experimental other treatments (Fig 3C). The C/N ratio of LM-inter-m in the 1.5MNPK and that of LM-inter-SC in the NPK were significantly (*P < 0*.*05*) lower than the control at 10–20 and 20–40 cm soil depths (Fig 3C and 3F). Among < 250 μm particles in LM fractions, the C/N ratio of LM-inter-SC was lower than that of LM-inter-m, and the C/N ratio of LM-inter-SC was in the range of 6.4–8.2.

**Fig 4:**
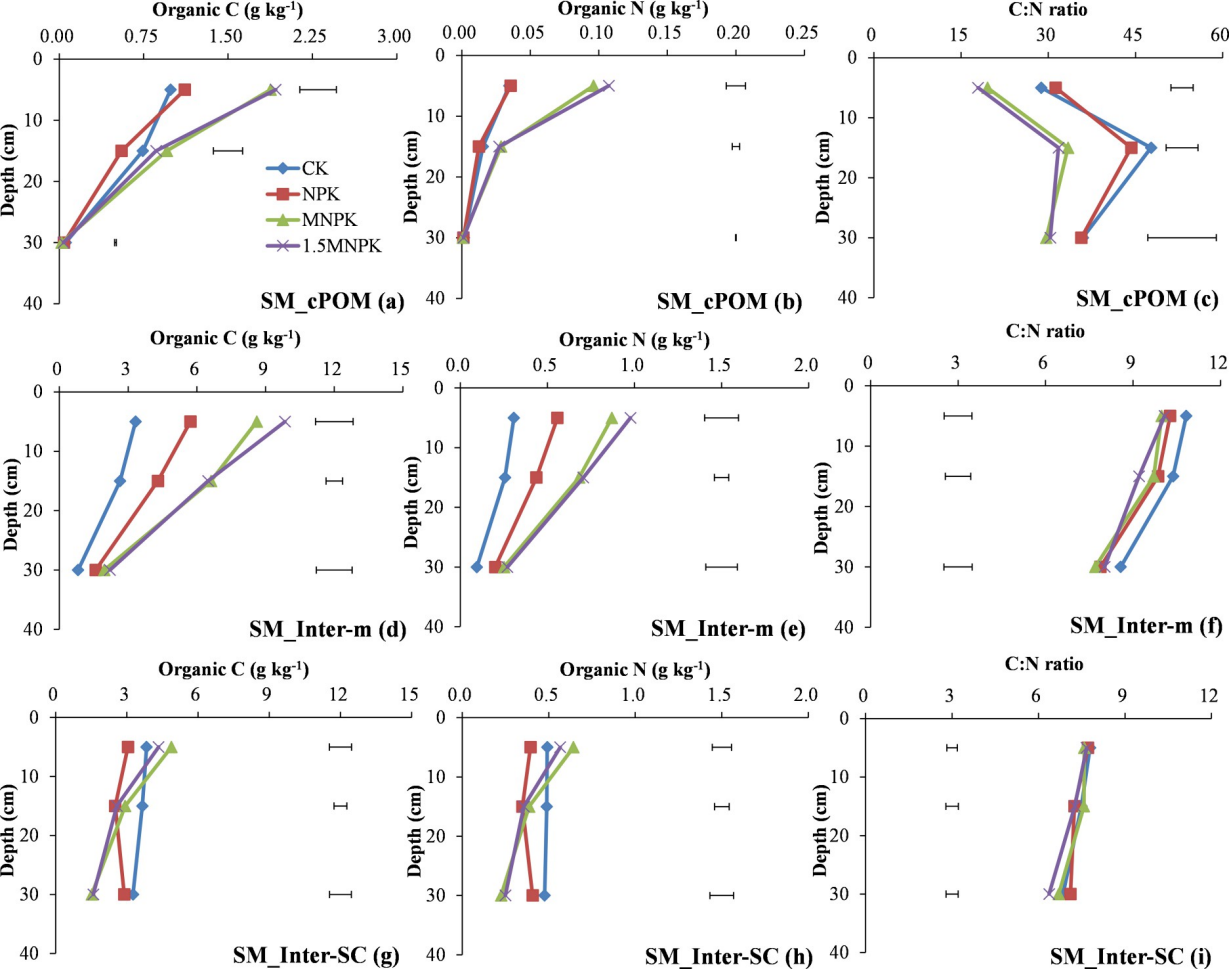
Change in organic C and N concentrations and their C/N ratios in small macroaggregate (SM) fractions of control (CK), chemical fertilizer (NPK), and combined manure and chemical fertilizer (MNPK, 1.5MNPK) treatments in the top 40 cm of the soil profile. Values at each soil depth are the mean of three replicates. The horizontal lines denote the least significant difference values at the 0.05 level (LSD0.05) among different treatments at each soil depth. Symbols of aggregate fractions: see Fig 1 footnote.

At 0–10 cm depth, the C and N concentrations of SM-cPOM in the manure treatments was significantly (*P < 0*.*05*) higher than in the other two treatments (Fig 4A and 4B) and there was a significant (*P < 0*.*05*) difference in the C concentration of SM-inter-m between manure application and control (Fig 4D and 4E) and in the C and N concentrations of SM-inter-SC between manure application and NPK (Fig 4G and 4H).

At 10–20 cm depth, the manure treatments significantly (*P < 0*.*05*) increased the C and N concentrations of SM-cPOM and SM-inter-m over the NPK and control except for the C concentration of SM-cPOM between manure treatments and control (Fig 4A, 4B, 4D and 4E). The control had significantly (*P < 0*.*05*) higher C and N concentrations of SM-inter-SC than the fertilization treatments (Fig 4G and 4H).

At 20–40 cm depth the control treatment significantly (*P < 0*.*05*) increased the C and N concentrations of SM-cPOM over the manure application treatments and the N concentration of SM-inter-SC over the manure application treatments (Fig 4A, 4B, 4G and 4H).

The control had significantly (*P < 0*.*05*) higher C/N ratios of SM-cPOM than the MNPK and 1.5MNPK treatments at 0–10 and 10–20 cm soil depths (Fig 4C). The control had significantly (*P < 0*.*05*) higher C/N ratios of SM-inter-m at 10–20 cm depth (Fig 4F) and SM-inter-SC at 20–40 cm depth (Fig 4I). Among < 250 μm particles in SM fractions, the C/N ratio of SM-inter-SC was lower than the other two fractions, and the C/N ratio of SM-inter-SC was in the range of 6.4–7.8.

**Fig 5:**
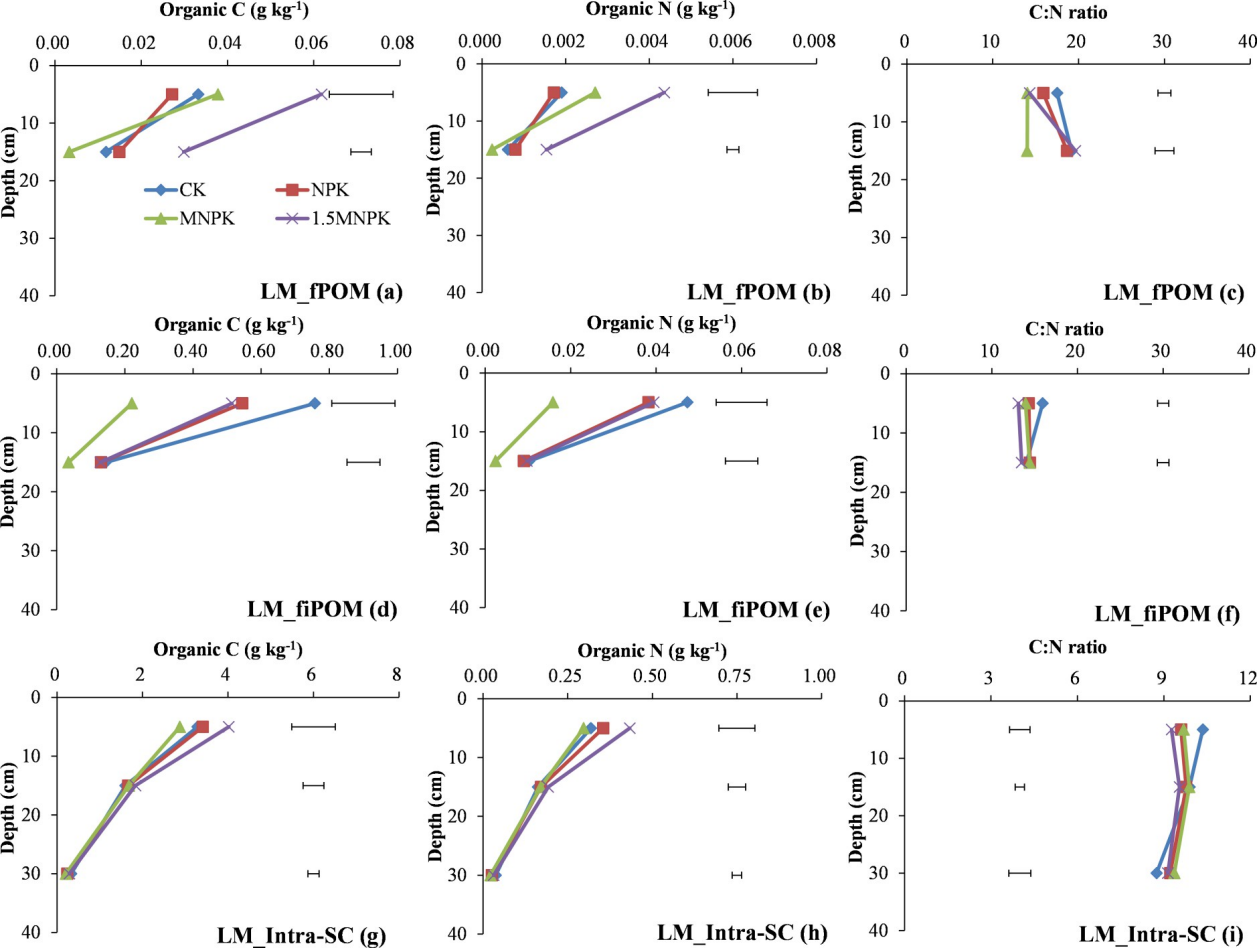
Change in organic C and N concentrations and their C/N ratios in microaggregates within large macroaggregate fractions of control (CK), chemical fertilizer (NPK), and combined manure and chemical fertilizer (MNPK, 1.5MNPK) treatments in the top 40 cm of the soil profile. Values at each soil depth are the mean of three replicates. The horizontal lines denote the least significant difference values at the 0.05 level (LSD0.05) among different treatments at each soil depth. Symbols of aggregate fractions: see Fig 1 footnote.

The C and N concentrations of LM-fPOM, LM-fiPOM, and LM-intra-SC in the MNPK were the lowest across treatments at each soil depth in most cases (Fig 5A, 5B, 5D, 5E, 5G and 5H). Moreover, there were significant (*P < 0*.*05*) differences in the C and N concentrations of LM-fPOM, LM-fiPOM, and LM-intra-SC between MNPK and 1.5MNPK at 0–10 cm depth, and significant (*P < 0*.*05*) differences in the C concentration of LM-fPOM and LM-fiPOM and the N concentration of LM-fPOM at 10–20 cm depth.

At 0–10 cm depth (Fig 5C, 5F and 5I), the C/N ratios of LM-fPOM, LM-fiPOM, and LM-intra-SC in the control were the highest among the four treatments, the C/N ratios of LM-fPOM and LM-fiPOM in the control were significantly (*P < 0*.*05*) higher than in the other treatments. At 10–20 cm depth, the C/N ratio of LM-fPOM in the MNPK was significantly (*P < 0*.*05*) lower than in the other three treatments. Among the four treatments, the averaged C/N ratio of LM-inter-SC was 1.57 units (-0.76–2.79 units) lower compared with free-SC or LM-intra-SC (Figs 2I, 3F and 5I).

**Fig 6:**
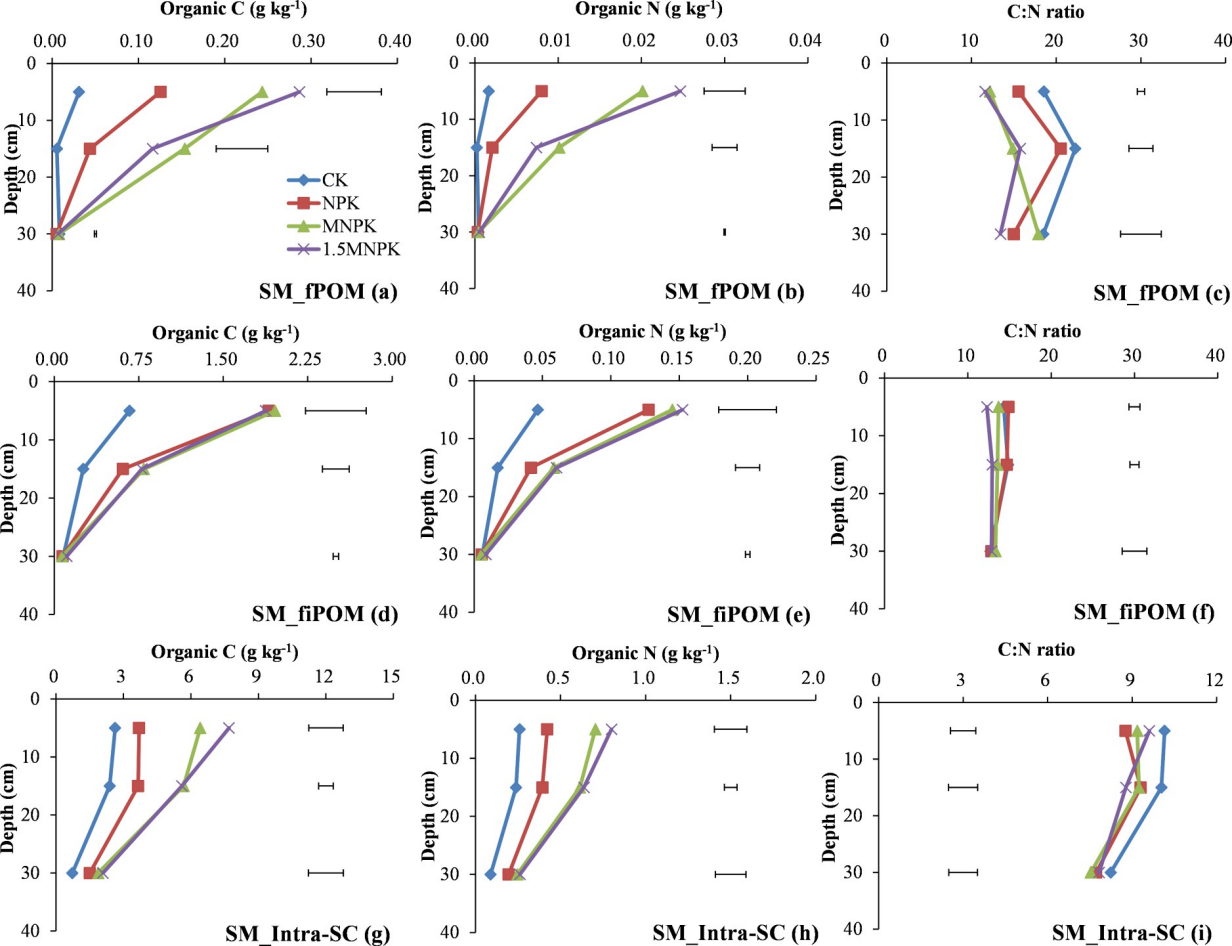
Change in organic C and N concentrations and their C/N ratios in microaggregates within small macroaggregate fractions of control (CK), chemical fertilizer (NPK), and combined manure and chemical fertilizer (MNPK, 1.5MNPK) treatments in the top 40 cm of the soil profile. Values at each soil depth are the mean of three replicates. The horizontal lines denote the least significant difference values at the 0.05 level (LSD0.05) among different treatments at each soil depth. Symbols of aggregate fractions: see Fig 1 footnote.

At 0–10 cm depth, manure application treatments had significantly (*P < 0*.*05*) higher C and N concentrations of SM-fPOM and SM-intra-SC than the other two treatments (Fig 6A, 6B, 6G and 6H), and the control had significantly (*P < 0*.*05*) higher C and N concentrations of SM-fiPOM than the other treatments (Fig 6D and 6E). At 10–20 cm depth, manure treatments had significantly (*P < 0*.*05*) higher C and N concentrations of SM-fPOM than the other two treatments (Fig 6A and 6B), and the fertilization treatments had significantly (*P < 0*.*05*) higher C and N concentrations of SM-fiPOM than the control (Fig 6D and 6E). At 20–40 cm depth, the control had significantly (*P < 0*.*05*) higher C concentration of SM-fPOM than the NPK treatment (Fig 6A and 6B).

The control and NPK treatments had significantly (*P < 0*.*05*) higher C/N ratios of SM-fPOM, SM-fiPOM, and SM-intra-SC than those of MNPK or 1.5MNPK at both 0–10 and 10–20 cm depths (Fig 6C, 6F and 6I). Among the four treatments, the averaged C/N ratio of SM-inter-SC was 1.46 units (-0.22–2.97 units) lower compared with free-SC or SM-intra-SC (Figs 2L, 4I and 6I).

**Fig 7:**
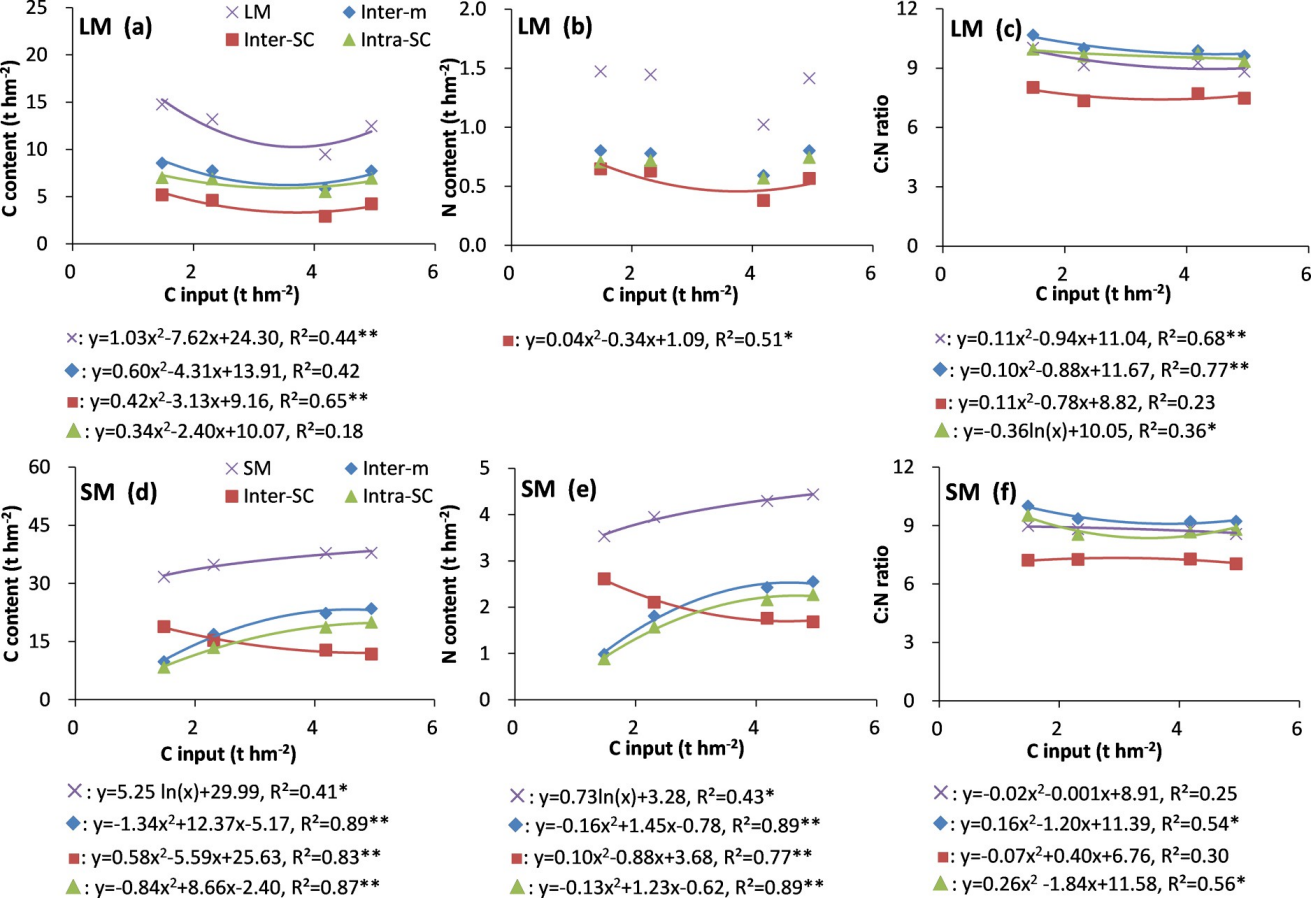
Relationships between C inputs and C and N stocks and their C/N ratios in POM, selected large and small macroaggregate fractions in the top 40 cm of the soil profile of the different treatments. Values are the mean of three replicates. C input sequence on the x-axis is CK, NPK, MNPK, and 1.5MNPK treatments, respectively; * and **, *P < 0*.*05* and *P < 0*.*01*; R^2^ is calculated using all replicates of the treatments. Symbols of aggregate fractions: see Fig 1 footnote.

In the LM fractions, there was a significant (*P < 0*.*05*) quadratic negative relationship between annual C input and organic C stock of LM, inter-SC and between annual C input and organic N stock of inter-SC (Fig 7A and 7B). In the SM fractions, the C and N stocks of SM, inter-m, and intra-SC showed an increasing trend with increasing annual C input, and the C and N stock of inter-SC showed a decreasing trend with increasing annual C input (Fig 7D and 7E). The SM showed significant (*P < 0*.*05*) logarithmic positive regression between C, N stock and annual C input rate (Fig 7D and 7E). Inter-m, inter-SC, and intra-SC in SM showed significant (*P < 0*.*05*) quadratic relationships between C, N stock and annual C input rate (Fig 7D and 7E). The C/N ratios of LM, LM-inter-m, SM-inter-m, and SM-intra-SC showed significant (*P < 0*.*05*) quadratic regressions with annual C input rate, and that of LM-intra-SC showed a significant (*P < 0*.*05*) logarithmic regression with annual C input rate (Fig 7C and 7F).

## Discussion

### Carbon and N concentrations in bulk soil

Soil organic C and N concentrations increased with increasing C input rate (Table 1; Fig 1A and 1B) because the increased C input provided more C sources for microbial N immobilization [7]. In the control and the chemical fertilizer treatment, the C input was derived mainly from crop residues and root C deposition; but in the manure application treatments the manure was an additional important C and N source for accumulation of soil organic C and N [7]. The concentrations of SOC and SON at 0–10 and 10–20 cm depths were therefore maximum in the 1.5MNPK treatment and minimum in the control (Fig 1A and 1B). The decline in SOC and SON concentrations in each treatment with increasing soil depth was due to the C input from the crop or manure remaining largely near the soil surface and the subsurface C input decreased distinctly, Peng et al. [28] also reported that root biomass decreased with increasing soil depth.

### Carbon and N concentrations and mass in aggregates

The physical protection of SOC in aggregates decides SOC formation and persistence, and SOC protected within aggregates is sensitive to soil management practices [8, 14]. Carbon sources from crop residues and manure provided energy for microorganism and promoted the mineral particles and small aggregates to form macroaggregates [8, 29]. Crop roots are an important binding agent in macroaggregate formation [30]. The higher root/shoot ratio required more available N to meet crop N demand where no N was applied, and root/shoot ratio decreased once the N rate exceeded crop N demand. Therefore, at 0–10 cm soil depth the LM mass values were significantly (*P < 0*.*05*) higher in the control and NPK treatments than in the other treatments. Furthermore, there was sufficient supply of C and N in the 1.5MNPK treatment, which resulted in a significant increase in LM mass than in the MNPK treatment (S1 Table in S1 File).

Macroaggregates represent complex mixtures of the labile and partially stable pools, macroaggregates formation and microaggregates binding into macroaggregates are regarded as an increase in SOM content [31, 32]. The large amounts of coarse POM and decomposable POM in the manure treatments resulted in an increase in microbial activity and binding agent production [33] as evidenced by the significant (*P < 0*.*05*) increases in C and N concentrations and the mass values in SM and its fractions in the manure treatments compared with the other treatments at 0–10 or 10–20 cm soil depth (S1 Table in S1 File, Fig 2A and 2B). Tillage in agricultural ecosystems can break down macroaggregates into smaller particles and thereby expose SOM in macroaggregates which was previously inaccessible to microbial attack [12, 13, 34]; the pores in microaggregates are much smaller and the organic matter in microaggregates is more recalcitrant than that in macroaggregates [30]; in addition, roots grow predominantly in the surface soil, the three reasons above explained that the SM mass across the four treatments at 0–10 cm depth was lower than that at 10–20 and 20–40 cm depths (S1 Table in S1 File), with the opposite trend in the LM mass. The C and N concentrations in LM and SM and their fractions were higher at 0–10 cm depth than at lower depths (plots a, b, d, e in Figs 2 & 3; plots a, b, d, e, g, h in Figs 4–6) in which there was an adequate SOM content near the soil surface for sequestration into inter-m because inter-m was the preferential C sequestration soil fraction when soil C inputs increased [8].

Particulate organic matter is an important binding agent in macroaggregates and an available index of the soil labile pool [35, 36]. Coarse POM contains the least decomposable materials and is closely related to the quantity and quality of C inputs and the eventual decomposition of cPOM forms fPOM and fiPOM [9, 33]. Therefore, manure application significantly (*P < 0*.*05*) increased the C and N concentrations of cPOM and fiPOM over the other treatments down to 20 cm depth (Fig 4A and 4B & plots d, e in Figs 5 and 6). The fPOM and fiPOM occur mainly in the microaggregates within macroaggregates and they would have made a considerable contribution to the increase in SOC stocks [15], although fiPOM was regarded as a component of the stable pool by Brown et al [9]. Here, most of the fiPOM across the four treatments had > 10 times the C and N concentrations and masses as fPOM at 0–10 cm soil depth (plots a, b, d, e in Figs 5 and 6; S1 Table in S1 File), this may be attributable to (1) the fiPOM with a density flotation of 2.35 g cm^-3^ being the stable sorption site of organic compounds associating with mineral particles against microbial degradation [37] and (2) fPOM being more easily subjected to microbial decay than fiPOM because of the repeated tillage [31, 38]. Additionally, the occurrence of the highest C and N concentrations and mass of LM-fiPOM in the control at 0–10 cm depth (Fig 5D and 5E; S1 Table in S1 File) further indicated that N deficiency promoted a close association between root biomass and soil particles to form LM.

In contrast to soil labile pool, soil stable pool is responsible for SOC storage and stabilization [34–36]. The free-m and the inter-m fractions were the main fractions in the stable pool, and both free-m and inter-m can serve as indicators of C sequestration. Furthermore, the sequestered C promotes microbial N immobilization [8, 9, 31]. These C inputs from crop residues or manures bind microaggregates or mineral particles into macroaggregates and enhanced aggregates development and protected SOM accumulation against microbial C and N mineralization [12, 13, 39]. Therefore, the C and N concentrations in the stable pool (S1d, S1e Fig in S1 File) as well as the C and N concentrations in SM-inter-m and SM-intra-SC significantly increased with increasing fertilization rate (Fig 4D, 4E, 4G and 4H). However, the control had the highest C and N concentrations in LM-inter-SC at 0–10 cm soil depth and SM-inter-SC at 10–20 and 20–40 cm soil depths (Figs 3D, 3E, 4G and 4H), and this may be due to a lack of available nutrients in the control promoting root biomass deeper in the soil profile to meet the nutrient demands of the crop. Peng et al. [28] also report that a control treatment had a higher root biomass in the deeper soil than chemical fertilizer treatments.

Soil particles in different fractions were closely associated and could be transferred between different size class aggregates during soil aggregation and disaggregation. The C and N concentrations in free-m, SM, and SM fractions were significantly (*P < 0*.*05*) negatively correlated with those in LM and LM fractions (S2a, S2b Fig in S1 File) indicating that free-m or SM can form LM and LM factions through binding agents from exogenous C sources or microbial residues and vice versa [15, 40]. Contrary or inconsistent correlation of aggregate fractions between C and N concentrations, for example, the C and N concentrations in LM vs. SM and SM-fPOM (Fig 6A and 6B) may be due to the interactions between organic C mineralization by N stimulation and N immobilization induced by available C sources. Overall, C and N storage in different size class aggregates is very complex.

The amount and capacity of SOM associated with soil particles are important factors influencing C and N sequestration [14]. The significant (*P < 0*.*05*) quadratic or logarithmic functions of the C and N contents in the LM and SM fractions with C input increased down to 40 cm depth (Fig 7A, 7B, 7D and 7E), indicating that C and N sequestration here occurred mainly in SM. Moreover, the inter-m in SM and intra-SC in inter-m were the main soil fractions involved in C and N sequestration in SM, and the SM-intra-SC further bound to SM-inter-m with increasing C input. Some studies report that microaggregates within macroaggregates are the dominant aggregate class accumulating C stocks [8, 9]. Even so, the soil C and N sequestration potential in soil aggregates can reach a plateau with increasing C input as shown by the C and N contents in SM, SM-inter-m, and SM-intra-SC (Fig 7D and 7E).

### C/N ratio

Soil C/N ratio is a major soil quality indicator but the C/N ratio in bulk soil is generally constant and hides the characteristics of the inherent C and N in soil [20] as shown by the lack of significant differences in C/N ratio among the different treatments at each soil depth (Fig 1C). Piñeiro et al. [20] reports that the C/N ratio of soil pools reflects the N availability in passive pools rather than the bulk soil. Here, the C/N ratio of LM was negatively correlated with those of SM, free-m, and free-SC (S2c Fig in S1 File), this may be due to much more C being associated with macroaggregates than with the smaller aggregates, with the opposite pattern of N in macroaggregates [41]. Furthermore, sufficient exogenous C provided the binding agent for the smaller aggregates to associate into macroaggregates. The positive correlation in C/N between LM and most of the soil particles and the negative correlation between SM and the particles was likely due to LM had more pores and sand than SM. When crop roots entered SM particles, soil particles in SM were more likely to encounter anaerobic conditions than those in LM in the wheat-rice rotation. For example, the C/N ratios of cPOM and fPOM in SM increased from 0–10 to 10–20 cm soil depth (Fig 6C and 6F) and the C/N ratio of fPOM in SM in the NPK and control treatments was -0.3 to 3.1 units larger than those in LM at 0–10 and 10–20 cm soil depths (Figs 5C and 6C). Across all the soil depths, the slight decrease in C/N ratio of bulk soil and all the aggregate fractions except for POM may be due to the 0–10 cm soil depth receiving more C inputs from manure or crop residues than the other soil depths (Figs 1–6).

The C/N ratio of POM reflects the original C/N ratio of exogenous organic material and the decomposable degree of different POM fractions [9, 33] as shown by the decline in C/N ratio from cPOM to fiPOM via fPOM in our study (Fig 4C; plots c & f in Figs 5, 6). The much higher C/N ratio of POM than of bulk soil indicates that POM is the main force driving soil aggregate C and N turnover [42]. Comparing the different POM fractions (Figs 3C, 4C and 4F), the highest C/N ratio in the control and the lowest C/N in the manure application treatments may be due to the control receiving no fertilizer N and the manure generally having a lower C/N ratio than the plant residues. At 10–20 cm soil depth (Figs 4C and 6C) the C/N ratio of cPOM and fPOM in SM had showed a clear increase that may be related to the higher organic C mineralization of POM and microbial N immobilization at 0–10 cm soil depth and the more prevalent anaerobic conditions at 10–20 cm depth. Throughout the top 40 cm of the soil profile the decrease in C/N ratio of LM and SM fractions with increasing C rate (Fig 7C and 7F) may be closely linked with the C/N ratio of exogenous substrates, that is, the manure had a much lower C/N ratio than the crop residues, and the applied C in control and the NPK treatment was derived mainly from crop residues.

The C/N ratios of the aggregate fractions making up the stable pool were the stabilizer of the bulk soil C/N ratio because their C/N ratios were similar to that of the bulk soil, and the C and N concentrations in these fractions dominated the bulk soil (Figs 1–7). In most cases at 0–10 and 10–20 cm soil depths (Figs 2–7), the C/N ratio in the fractions of the soil stable pool in the control had the highest C/N ratio because of N deficiency resulting in crop residues with high C/N ratio, and the C/N ratios of these soil fractions decreased with increasing C and N rate. However, the change in C/N ratios with increasing C and N rates were inconsistent. For example, the significantly (*P < 0*.*05*) highest C/N ratio of free-SC in the 1.5MNPK treatment at 20–40 cm soil depth (Fig 2L) may have been due to the excessive dissolved organic matter leached and then adsorbed by free-SC. The second most significantly (*P < 0*.*05*) higher C/N ratio of LM-intra-SC in MNPK (Fig 5I) or SM-intra-SC in the 1.5MNPK treatments at 0–10 cm soil depth (Fig 6I) may have been due to intra-SC being the main particle involved in C storage in microaggregates within macroaggregates in the long term with manure application promoting SOC accumulation [8].

Different with our second hypothesis, the inter-SC in LM and SM (Figs 3F and 4I) may have been the main soil particle involved in N immobilization in macroaggregates because (1) the occlusion degree of inter-SC was intermediate between free-SC and intra-SC according to aggregate hierarchy theory, but the C/N ratio of inter-SC in LM and SM was lower compared with free-SC or intra-SC in LM and SM (Figs 2I, 5I and 6I); (2) the sand in silt and clay particles decreased with increasing occlusion degree of silt-clay particles [14], resulting in decreased oxygen in the higher occluded < 53 μm soil particles. Correspondingly, the higher C concentration was protected in the intra-SC fraction across soil depths in each treatment (Fig 4G); and (3) the sorption and desorption of soil mineral nutrients was more prominent on fine fractions than on coarse fractions [12, 13].

## Conclusions

The results here indicate that the formation or fragmentation of soil aggregates was the comprehensive result of crop roots, soil tillage and fertilization practices. Small macroaggregates were the dominant sequestrated C soil particles as shown by the significant (*P < 0*.*05*) increase in the mass and the C and N concentrations in SM in the manure treatments than the other treatments at 0–10 cm soil depth, and the C and N contents down to 40 soil depth in SM and its fractions tended to saturation in the 1.5MNPK treatment. Long-term manure application was an effective practice for increasing C and N concentrations and decreasing the C/N ratios in the bulk soil and different size class aggregates down to 20 cm soil depth in most cases, the lower C/N ratios of inter-SC among all the silt-clay particles indicated that the inter-SC was the dominant particle involved in soil N immobilization. Overall, with the improvement of soil structure after long-term manure application in the wheat-rice cropping system, SM was the main and preferential aggregates for C and N stock, moreover, inter-SC had more N availability than the other soil particles among all the aggregates fractions.

## Supporting information

S1 Data (XLSX)

S1 File (PDF)
