# Correction: Long-term combined organic manure and chemical fertilizer application enhances aggregate-associated C and N storage in an agricultural Udalfs soil

**DOI:** 10.1371/journal.pone.0315524

**Published:** 2024-12-05

**Authors:** Shaojun Qiu, Cheng Hu, Donghai Liu, Shuanglai Li, Shicheng Zhao, Xinpeng Xu, Ying Zhao, Ping He, Wei Zhou

The third author’s name is spelled incorrectly. The correct name is: Shuanglai Li. The correct citation is: Qiu S, Hu C, Liu D, Li S, Zhao S, Xu X, et al. (2023) Long-term combined organic manure and chemical fertilizer application enhances aggregate-associated C and N storage in an agricultural Udalfs soil. PLOS ONE 18(2): e0276197. https://doi.org/10.1371/journal.pone.0276197.
