# Supplementary material for: Long-term combined organic manure and chemical fertilizer application enhances aggregate-associated C and N storage in an agricultural Udalfs soil
Source: PLoS One. 2023 Feb 13;18(2):e0276197. doi: 10.1371/journal.pone.0276197 (PMC9925001; doi:10.1371/journal.pone.0276197)
Supplement: S1 File — (PDF) [file pone.0276197.s002.pdf]

Supporting information

Long-term combined organic manure and chemical fertilizer application enhances aggregate-associated C and N storage in an agricultural Udalfs soil

Shaojun Qiu[a], Cheng Hu[b], Donghai Liu [b], Shuanglai Liu[b], Shicheng Zhao[a], Xinpeng Xu[a], Ying Zhao[c], Ping He [a], Wei Zhou [a]

Results

SM was the largest WSA fraction across all four treatments and three soil depths (Table S1). Manure application significantly ($P < 0.05$) decreased LM and significantly ($P < 0.05$) increased SM over the other two treatments at 0-10 cm soil depth and the NPK treatment at 10-20 cm soil depth. MNPK significantly ($P < 0.05$) increased free-m over the other three treatments at 0-10 cm soil depth, free-SC at 0-10 cm depth and free-m at 20-40 cm depth compared with the 1.5MNPK treatment.

In the LM fractions at 0-10 and 10-20 cm depths (Table S1) the mass values of inter-m, inter-SC, and intra-SC in one or both manure application treatments were significantly ($P < 0.05$) lower than that in the other two treatments; fiPOM in the MNPK treatment was significantly ($P < 0.05$) lower than in the other treatments.

In the SM fractions (Table S1) manure application gave significantly ($P < 0.05$) higher mass values of cPOM and fPOM at 0-10 cm soil depth, fPOM and fiPOM at 10-20 cm soil depth, and inter-m and intra-SC at 10-20 and 20-40 cm soil depths compared to the other two treatments. Fertilization treatments significantly ($P < 0.05$) decreased

the inter-SC at 10-20 and 20-40 cm soil depths compared to the control. NPK gave significantly ($P < 0.05$) higher fPOM, fiPOM, inter-m, and intra-SC than the control at 10-20 cm depth, and the control had significantly ($P < 0.05$) higher inter-SC at each soil depth down to 40 cm.

Table S1

The manure treatments had significantly ($P < 0.05$) higher C and N concentrations in labile and stable pools than the other two treatments at 0-10 and 10-20 cm depths (Fig. S1a, b, d, e). Moreover, 1.5MNPK had significantly higher ($P < 0.05$) the N concentrations of labile and stable pools as well as C concentrations of stable pools than MNPK at 0-10 cm depth (Fig. S1a, b, d, e). Conversely, the control had significantly ($P < 0.05$) higher C and N concentrations in the labile pool than the other treatments at 20-40 cm depth (Fig. S1a, b).

The manure application treatments had a significantly ($P < 0.05$) lower labile pool C/N ratio than the other two treatments at each soil depth (Fig. S1c). The control had a significantly ($P < 0.05$) higher stable pool C/N ratio than MNPK at 0-10 cm depth and labile pool C/N ratio than the other three treatments at 10-20 cm depth (Fig. S1c, f).

Fig. S1

The C concentrations in the LM or SM fractions were usually significantly ($P < 0.05$) positively correlated with their own fractions (Fig. S2a). A significant ($P < 0.05$) negative correlation in C concentration was found between SM and LM as well as

between most SM fractions and most LM fractions (Fig. S2 a). The C concentrations in free-m, free-SC, labile pool and stable pool had significant ($P < 0.05$) positive correlations with some of the SM fractions and significant ($P < 0.05$) negative correlations with some of the LM fractions (Fig. S2 a). The same correlation relationships were found in N concentrations (Fig. S2 b) as in C concentrations in the different size categories of aggregates (Fig. S2 a). The free-m N concentration was significantly ($P < 0.05$) positively correlated with the free-SC N concentration (Fig. S2 b).

Significant ($P < 0.05$) negative correlations in C/N ratio were found between LM and SM in the different size classes of aggregates (Fig. S2 c). The LM C/N ratio was significantly ($P < 0.05$) positively correlated with the LM and SM fractions, and the SM C/N ratio was significantly ($P < 0.05$) negatively correlated with the LM and SM fractions. The C/N ratio of free-m was significantly ($P < 0.05$) negatively correlated with SM, and LM_fPOM and significantly ($P < 0.05$) positively correlated with SM_free-SC. The C/N ratio of free-SC was significantly ($P < 0.05$) negatively correlated with SM_cPOM, SM_fiPOM and LM_fPOM, and positively correlated with SM_inter-SC. The C/N ratio of SM_cPOM was significantly ($P < 0.05$) positively correlated with LM_fPOM and SM_fPOM and significantly ($P < 0.05$) negatively correlated with SM_inter-SC. A significant ($P < 0.05$) positive correlation in C/N ratio was found between LM_fPOM and SM_fPOM, between SM_fPOM and SM_fiPOM, and between inter-m and intra-SC in LM or SM.

The C/N ratio of the labile pool was significantly ($P < 0.05$) positively correlated

with those of LM, cPOM and fiPOM in SM and with fPOM in LM and SM as well as was significantly ($P < 0.05$) negatively correlated with those of free-SC and SM_inter-SC (Fig. S2 c). The stable pool showed the opposite trends and the stable pool C/N ratio was significantly ($P < 0.05$) positively correlated with the C/N ratios of free-m and SM_inter-m and negatively with the C/N ratio of the labile pool.

Fig. S2

Table S1 Water stable aggregate mass as well as large and small macroaggregate fractions mass among control (CK), chemical fertilizer (NPK), and the combination of manure and chemical fertilizer (MNPK, 1.5MNPK) treatments in the top 40 cm of the soil profile. Unit: %

	Water stable aggregates (WSA)				Large macroaggregates (LM)		Small macroaggregates (SM)			Density fractionation					
										LM			SM		
Treatment	LM	SM	Free-m	Free-SC	Inter-m	Inter-SC	cPOM	Inter-m	Inter-SC	fPOM	fiPOM	Intra-SC	fPOM	fiPOM	Intra-SC
0-10cm															
CK	40.2a	46.3b	9.5b	2.6a	17.8a	20.8a	0.40b	12.6b	31.4a	0.017bc	0.31a	17.1a	0.02c	0.26b	12.1b
NPK	37.6a	48.7b	10.1b	2.0ab	17.6a	17.6a	0.46b	24.0a	22.8b	0.015c	0.25a	16.7a	0.10b	0.90a	22.8a
MNPK	21.2c	59.6a	15.1a	2.4a	10.7b	8.6c	0.97a	27.3a	29.1ab	0.025ab	0.08b	10.2b	0.20a	1.10a	25.8a
1.5MNPK	30.2b	56.2a	10.4b	1.6b	14.3ab	13.0b	1.06a	27.9a	24.7ab	0.032a	0.23a	13.8ab	0.22a	0.97a	26.1a
10-20cm															
CK	21.7a	58.5ab	15.4a	3.0a	10.7a	9.7a	0.24bc	15.6c	41.0a	0.009b	0.09a	10.4a	0.003d	0.10c	15.1c
NPK	21.1a	54.9b	18.5a	3.3a	11.0a	9.5ab	0.18c	27.4b	26.1b	0.013a	0.07a	10.4a	0.03c	0.27b	26.6b
MNPK	15.2b	60.9a	18.1a	3.6a	7.7b	6.2c	0.35a	32.9a	26.1b	0.003c	0.02b	7.5b	0.15a	0.33a	31.4a

1.5MNPK	16.2b	61.9a	16.6a	2.7a	8.0b	7.4bc	0.30ab	35.9a	24.1b	0.012ab	0.07a	7.7b	0.08b	0.34a	34.7a
20-40cm															
CK	7.9a	62.0a	25.7ab	3.9c	3.9a	3.4ab	0.028a	9.1b	51.2a	0.003a	0.05	3.8a	0.007b	0.036a	8.8b
NPK	4.9c	56.8b	28.5a	8.2a	2.4a	2.3b	0.015b	14.0b	41.9b	0.002ab	-	-	0.009b	0.039a	13.8b
MNPK	5.1bc	60.7ab	27.7a	5.9b	2.6a	1.8b	0.014b	27.2a	31.4c	0.001b	-	-	0.013a	0.055a	26.5a
1.5MNPK	7.7ab	63.8a	21.8b	4.7bc	3.1a	4.0a	0.016b	30.7a	31.1c	0.004a	-	3.1a	0.007b	0.056a	30.0 a

Note:

Values at each depth are shown the mean of three replicates. Different letters in the same column denote the least significant difference values at the 0.05 level (LSD0.05) among different treatments at each depth. “-”, sample wasn’t separate from soil.

LM, SM, free-m, and free-SC represent large macroaggregates (> 2000μm), small macroaggregates (2000 - 250 μm), microaggregates (53-250 μm), and silt-clay (< 53 μm); cPOM, inter-m, and inter-SC represent coarse particulate organic matter (POM), microaggregates within macroaggregates, and silt-clay within macroaggregates; fPOM, fiPOM, and intra-SC represent fine POM, fine intra POM and intra silt-clay within inter-m.

Figure Titles

Fig. S1 Change in organic C and N concentrations and their C/N ratios in labile and stable fractions of chemical fertilizer (NPK) and combined manure and chemical fertilizer (MNPK, 1.5MNPK) treatments in the top 40 cm of the soil profile.

C or N concentration in labile fraction is the sum of C or N concentration in cPOM and fPOM; correspondingly, that in the stable fraction is the sum in free-m, free-SC as well as fiPOM, inter-SC and intra-SC in large and small macroaggregates.

Values at each soil depth are the mean of three replicates. The horizontal lines denote the least significant difference values at the 0.05 level (LSD0.05) among different treatments at each soil depth. Symbols of aggregate fractions: see Table S1 footnote.

Fig. S2 Partial correlations of C and N concentrations and C/N ratios of soil aggregate particles controlling soil organic C and N contents and C/N ratios in bulk in the top 20 cm of the soil profile. Values of soil aggregate particles at 20-40 cm soil depth are omitted because some values of soil aggregate particles were not detected. *, **, and *** indicate significance at $P < 0.05$, $P < 0.01$, and $P < 0.001$.

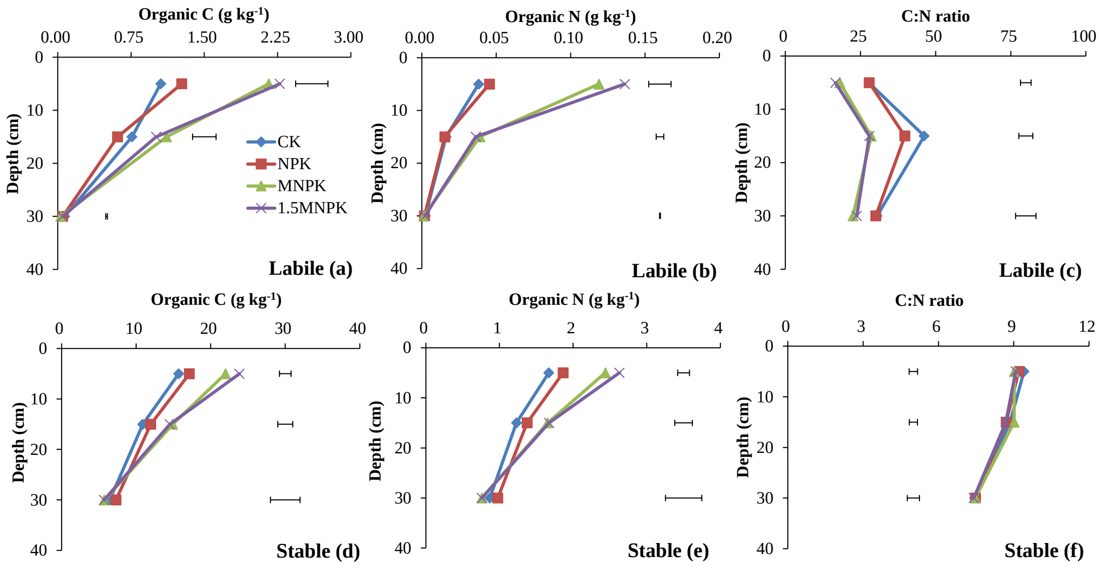
Organic C (g kg-1)
Organic N (g kg-1)
C:N ratio
Depth (cm)
CK
NPK
MNPK
1.5MNPK
Labile (a)
Labile (b)
Labile (c)
Stable (d)
Stable (e)
Stable (f)

Fig. S1

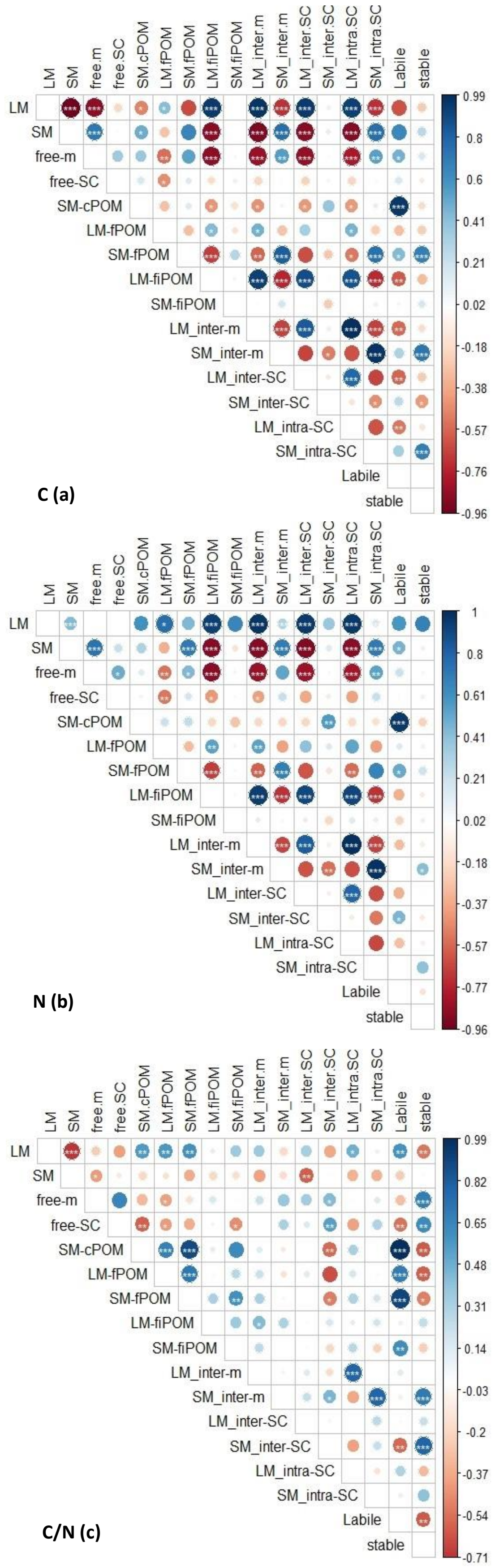
LM
SM
free.m
free.SC
SM.cPOM
LM.fPOM
SM.fPOM
LM.fiPOM
SM.fiPOM
LM_inter.m
SM_inter.m
LM_inter.SC
SM_inter.SC
LM_intra.SC
SM_intra.SC
Labile
stable
LM
SM
free-m
free-SC
SM-cPOM
LM-fPOM
SM-fPOM
LM-fiPOM
SM-fiPOM
LM_inter-m
SM_inter-m
LM_inter-SC
SM_inter-SC
LM_intra-SC
SM_intra-SC
Labile
stable
0.99
0.8
0.6
0.41
0.21
0.02
-0.18
-0.38
-0.57
-0.76
-0.96
C (a)
1
0.8
0.61
0.41
0.21
0.02
-0.18
-0.37
-0.57
-0.77
-0.96
N (b)
0.99
0.82
0.65
0.48
0.31
0.14
-0.03
-0.2
-0.37
-0.54
-0.71
C/N (c)

Fig. S2
